# Novel PCB-degrading *Rhodococcus* strains able to promote plant growth for assisted rhizoremediation of historically polluted soils

**DOI:** 10.1371/journal.pone.0221253

**Published:** 2019-08-22

**Authors:** Lorenzo Vergani, Francesca Mapelli, Jachym Suman, Tomas Cajthaml, Ondrej Uhlik, Sara Borin

**Affiliations:** 1 Department of Food, Environmental and Nutritional Sciences, University of Milan, Milan, Italy; 2 Departement of Biochemistry and Microbiology, University of Chemistry and Technology Prague, Prague, Czech Republic; 3 Institute of Microbiology, Czech Academy of Sciences, Prague, Czech Republic; Universidade do Porto, PORTUGAL

## Abstract

Extended soil contamination by polychlorinated biphenyls (PCBs) represents a global environmental issue that can hardly be addressed with the conventional remediation treatments. Rhizoremediation is a sustainable alternative, exploiting plants to stimulate *in situ* the degradative bacterial communities naturally occurring in historically polluted areas. This approach can be enhanced by the use of bacterial strains that combine PCB degradation potential with the ability to promote plant and root development. With this aim, we established a collection of aerobic bacteria isolated from the soil of the highly PCB-polluted site “SIN Brescia-Caffaro” (Italy) biostimulated by the plant *Phalaris arundinacea*. The strains, selected on biphenyl and plant secondary metabolites provided as unique carbon source, were largely dominated by Actinobacteria and a significant number showed traits of interest for remediation, harbouring genes homologous to *bphA*, involved in the PCB oxidation pathway, and displaying 2,3-catechol dioxygenase activity and emulsification properties. Several strains also showed the potential to alleviate plant stress through 1-aminocyclopropane-1-carboxylate deaminase activity. In particular, we identified three *Rhodococcus* strains able to degrade *in vitro* several PCB congeners and to promote lateral root emergence in the model plant *Arabidopsis thaliana in vivo*. In addition, these strains showed the capacity to colonize the root system and to increase the plant biomass in PCB contaminated soil, making them ideal candidates to sustain microbial-assisted PCB rhizoremediation through a bioaugmentation approach.

## Introduction

The remediation of soils polluted by polychlorinated biphenyls (PCBs) represents a major environmental challenge due to the worldwide spread of contaminated sites [1–5]. This family of xenobiotics presents high persistence in the environment, bioaccumulation through the food web and toxic effects on human being [6, 7]. The site “SIN (Site of National Priority) Brescia-Caffaro”, located in northern Italy (45° 32’ 8 N; 10° 12’ 52 E) and originated by the activity of the Caffaro PCB producing plant, includes more than 100 ha of former agricultural soils heavily polluted by metals and persistent chlorinated organic pollutants, with a prevalence of PCBs that often exceed both the residential (0.001 mg/Kg) and the industrial (5 mg/Kg) law limits [4, 8, 9]. Because of the high economic and environmental constrains, physical and chemical treatments are often not applicable to clean-up PCB-polluted sites presenting extended contaminations such as the SIN Brescia-Caffaro, so there is the necessity to develop low-cost strategies that can mitigate the xenobiotic concentration at the same time preserving the soil functionality [10–12]. In this context, rhizoremediation represents an *in situ* sustainable technology for the remediation of soil contaminated by PCBs, based on the use of plants to stimulate the soil degrading bacterial community [13–15].

Root exudates represent indeed an easily accessible carbon source for the soil microorganisms, potentially enhancing their metabolic activity in the rhizosphere. Rhizodeposits, moreover, can contain plant secondary metabolites (PSMs) that foster the bacterial ability to degrade PCBs through co-metabolism, and/or acting as biosurfactants increasing PCB bioavailability [16–19]. The efficiency of the rhizoremediation process may be increased by bioaugmentation with PCB-degrading microbial isolates. Autochthonous bioaugmentation is of major interest because it relies on strains already adapted to the ecological conditions of the site and prevents the introduction of potentially dangerous invader species [20].

Microorganisms, besides their degradation potential, can contribute to the efficiency of rhizoremediation also by plant growth promoting (PGP) traits. PGP microorganisms can increase the amount of contaminated soil explored by the plant by improving the architecture and extension of the plant root system and can counteract the possible phytotoxic effects of the polluted soil by decreasing the plant response to abiotic stresses [15, 21, 22]. Microbial isolates combining both the xenobiotic degradation and the PGP potential are in this frame a smart and sustainable tool to be exploited in the planning of rhizoremediation interventions. In this work, we established a collection of aerobic bacteria able to grow on biphenyl and PSMs as unique carbon sources from the soil of the SIN Brescia-Caffaro biostimulated by the plant species *Phalaris arundinacea* and subjected to a redox cycle for three-months during a greenhouse rhizoremediation experiment [23]. The redox cycle was applied to stimulate in anoxic conditions the reductive dechlorination of highly chlorinated PCB congeners, and in the subsequent aerobic conditions the oxidative biodegradation of the lower chlorinated ones, comprising the congeners originally present in the soil and the ones generated by the reductive degradation occurred in the anoxic phase [24]. We identified and characterized the bacterial isolates *in vitro* to assess their potential for i) biodegradation and ii) plant growth promotion, aiming to select the most promising strains to be exploited for autochthonous bioaugmentation in assisted PCB-rhizoremediation strategies at the SIN Brescia-Caffaro.

## Materials and methods

### Isolation of bacteria

Isolation of bacteria was performed from the root-surrounding soil [25] collected from three replicated pots planted with *Phalaris arundinacea* for three months and subjected to oxic-anoxic cycles by periodical flooding. The soil was collected destructively from each pot, homogenized and stored overnight at 4°C until the isolation of bacteria was performed [23]. One gram of soil was suspended in 9 ml of physiological solution (0.9% NaCl), diluted in ten-fold series and plated onto Petri dishes containing agar mineral medium [26] supplemented with biphenyl, limonene or naringin (SIGMA Aldrich) as unique carbon source. Biphenyl crystals were placed on the dishes lid, limonene was let evaporate from a vial inside a jar where Petri dishes were incubated and naringin was dissolved in ethanol and added to the autoclaved mineral medium to a final concentration of 2 g/L [27]. After one week of incubation at 30°C, 30 colonies for each replicate and each medium (n = 270 colonies) were randomly picked, taking into account the observed morphologies, and spread three times on mineral medium supplemented with biphenyl with the aim to select the strains able to metabolize this molecule, considered a proxy for PCB degradation [28–30]. One hundred twenty-eight isolates able to grow on biphenyl were kept for further characterization. The isolates were labelled with codes including numbers 1, 2, 3 and letters B, L, and N according to the sample replicate and the isolation medium (B = biphenyl, L = limonene, N = naringin), followed by a progressive number for each isolate.

### Bacterial identification

The DNA of each isolate able to grow on biphenyl was extracted through CTAB–phenol chloroform DNA extraction [31] and the collection was de-replicated using the ribosomal internal transcribed spacers (ITS)-PCR fingerprinting protocol [32]. One representative strain from each polymorphic ITS profile (n = 56) was identified through 16S rRNA gene amplification using primers 27F and 1492R followed by partial sequencing with primer 27F (Macrogen, Rep. of South Korea) as described by Mapelli et al. (2013). 16S rRNA nucleotide sequences were analysed for taxonomic identification using the EzBioCloud database [33]. The identification of the strains 3B12, 2B23 and 2B27, the most promising ones according to the functional characterization, was further confirmed through complete 16S rRNA gene sequencing with both forward and reverse primers. The sub-collection constituted by the identified strains was stored at -80°C in R2A liquid medium with 25% glycerol for further analysis.

### *BphA* gene amplification

The presence of the genes encoding for biphenyl dioxygenase α subunit (*bphA*) was assessed through PCR with primers 512F and 674R (obtaining an amplicon of 162 bp) according to Leewis et al. (2016) with the following protocol and conditions: 95°C (5 min), then 40 cycles of 95°C (30 s), 56°C (30 s) and 72°C (30 s), final elongation step 72°C (10 min); DNA template 2 μl, primers 1 μM, dNTPs 2 mM, polymerase 1,5 U per reaction. The sequence identity and diversity of *bphA* genes were further evaluated as described by [35] using BPHD-F3/R1 primer set amplifying a broader gene fragment of 542 bp. The PCR protocol was set up as follows: 95°C (3 min), then 30 cycles of 95°C (45 s), 60°C (45 s) and 72°C (40 s), final elongation step 72°C (4 min). All PCR reactions were performed utilizing FastStart High Fidelity PCR System (Roche). The DNA of the PCB-degrading strain *Paraburkholderia xenovorans* LB400 (DSMZ, Germany) was used as positive control to assess primer specificity and the amplicon sequenced to confirm identity with *bphA* gene. Amplicons were visualized on 1.2% agarose gel electrophoresis and PCR products that showed a single band of the predicted size were sequenced at Eurofins Genomics Srl (Italy). Nucleotide sequences were then analysed using the blastn suite to assess nucleotide percentage identity with known dioxygenases deposited in the NCBI database. Nucleotide sequences were deposited in the ENA database under accession numbers LT978340-LT978395 (16S rRNA gene) and LT986331-LT986350 (*bphA* gene).

### *In vitro* screening for traits of interest in assisted rhizoremediation

The same bacterial strains chosen for taxonomic identification as representative of each ITS profile (n = 56) among the isolates able to grow on biphenyl were also selected for *in vitro* functional screening aiming to test rhizoremediation potential within the collection. Catechol 2,3-dioxygenase activity was tested according to [36]. The isolates were grown in tryptic soy broth (TSB) medium for 48 hours at 30°C under orbital agitation, then 50 μl of the bacterial suspension were added to 150 μl of a catechol solution (catechol 90 mM, Tris-acetate buffer pH 7.5 50 mM) and incubated in the dark for two hours at room temperature. The result was considered positive if the samples developed a green-brownish colour compared to the cell-free negative control.

Biosurfactant production was evaluated by a whole cell emulsification test. Bacterial strains were grown in a test tube in TSB medium for 48 hours in agitation, and then mineral oil was added to the culture at a 1:1 ratio. The two phases were mixed by vortexing thoroughly for two minutes. After 30 minutes, the emulsification between the two phases was evaluated and measured according to [37] comparing it to a positive abiotic control containing 10% SDS. The stability of the emulsification was evaluated by incubating the test tubes for 48 hours at room temperature and comparing the height of the emulsification after 30 minutes and after 48 hours. The 1-aminocyclopropane-1-carboxylate (ACC) deaminase activity was tested following the protocol described by [38].

### *In vivo* PGP test on *Arabidospis thaliana* seedlings

**Fig 1:**
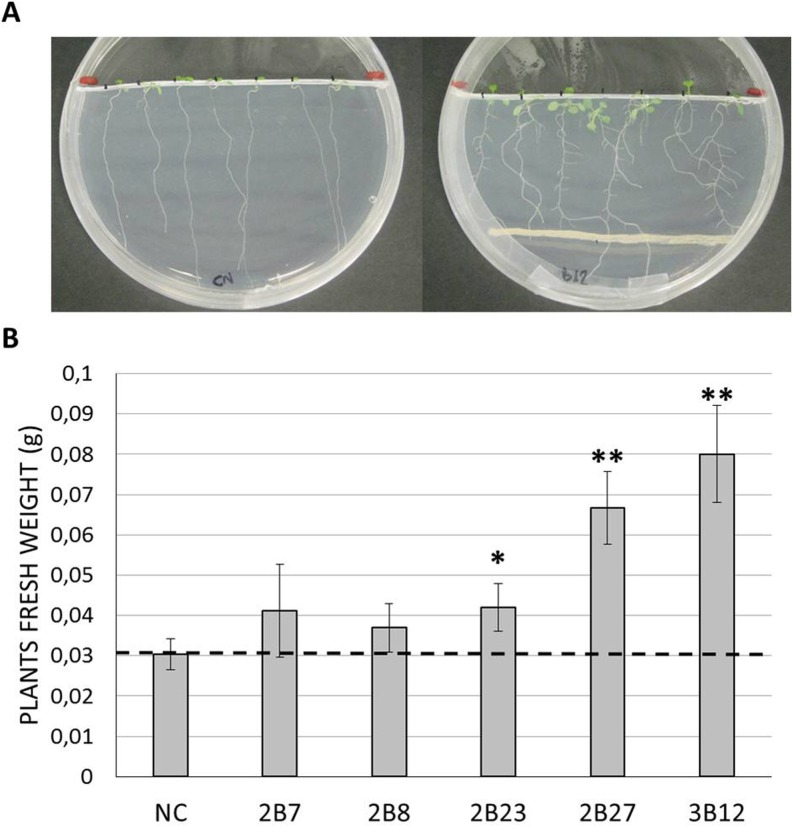
Plant growth promoting assay on *Arabidopsis thaliana*. **(A)** The pictures exemplify the promotion of lateral root development by *Rhodococcus jostii* strain B12 compared to the non-inoculated negative control (NC). **(B)** Screened strains and plant fresh weight measurement results are reported on the X-and Y-axes, respectively. The stars indicate statistically significant differences compared to the negative control (* = p<0.05, ** = p<0.01).

Only the bacterial strains displaying biphenyl dioxygenase gene amplification with both primer sets used in this study were selected for further characterization through *in vivo* PGP test. Plant growth promotion of *Arabidopsis thaliana* was assessed as follows: *A*. *thaliana* (ecotype Col-0) seeds were surface-sterilized by soaking in 70% ethanol for 2 minutes and in 1% sodium hypochlorite for 20 minutes. After five washing steps with sterile distilled water the seeds were placed on Petri dishes containing MS medium supplemented with sucrose (Murashige and Skoog basal salt mixture 2.15 g/L, sucrose 15 g/L, agar 8 g/L) and vernalized at 4°C in the dark for 72 hours. The plates were then transferred for seed germination into a growth chamber at 22°C, 50% relative humidity and a 16-h light/8-h dark exposure. After three days, *Arabidopsis* seedlings were transferred on fresh medium into plates prepared by severing 1/3 of the solid medium. Seven plantlets for each plate were disposed on the cut’s edge and the Petri dishes were incubated vertically in the growth chamber. The bacterial strains to be tested for plant growth promotion activity were streaked at the opposite side of the seedlings, in parallel to the cut (Fig 1A). Five replicated Petri dishes for each strain and for a non-inoculated control were set up, for 35 plantlets/thesis. Plant fresh weight was recorded after two weeks and a Student T-test was applied to perform a pairwise comparison between the plants inoculated with bacterial strains and the non-inoculated control plants, to assess the occurrence of statistically significant differences.

### Resting cell assay for PCB degradation assessment

As done for *in vivo* PGP activity, the bacterial strains positive to *bphA* amplification with both the primer sets used in this study were tested for PCB degradation. Bacterial strains were grown at 28°C on a rotatory shaker in mineral medium [26] supplemented with sodium pyruvate 30 mM and biphenyl crystals to induce the *bph* gene expression. A 1% inoculum was then transferred into flasks containing 250 ml of the same medium and incubated at the same conditions. The culture was filtered in sterile conditions using a funnel filled with glass wool to remove biphenyl crystals, washed two times with sterile physiological solution (0.9% NaCl) and suspended in mineral medium in order to obtain an optical density of 1 at 600 nm wavelength. A volume of 100 ml of each bacterial suspension was collected and autoclaved to set up negative controls. Four replicates for each strain and the respective negative control were set up as follows: 20 ml of the bacterial suspension were transferred into 100 ml serum bottles and spiked with 20 μl of 1% PCB, commercial mixture Delor 103 [39]. An additional negative control was set up by using the mutant strain *Pseudomonas alcaliphila* JAB1 having a disrupted *bph* operon [40]. The bottles were sealed and incubated for 48 hours on a rotatory shaker at 28°C, and then the reaction was stopped by freezing the bottles at -20°C. The content of individual PCB congeners present in the microcosms was determined using gas chromatography-mass spectrometry (GC-MS; 450-GC, 240-MS ion trap detector, Varian, Walnut Creek, CA). PCBs were analysed in ethyl acetate extracts according to the method described by [41]. Total PCB content was expressed for each microcosm as a sum of the individual congeners measured and PCB degradation ability was assessed by a statistical analysis (Student T-test) between each strain and its inactivated (*i*.*e*. autoclaved) control or the JAB1 control.

### PGP activity assay in PCB contaminated microcosms

The bacterial strains i) positive to *bphA* amplification with both primer sets used in this study and displaying ii) *in vivo* PGP activity and iii) *in vitro* PCB degradation ability were selected for further characterization in microcosm conditions. *A*. *thaliana* seeds (ecotype Col-0) were sterilized as described above, then soaked for 1 h at 25°C in a bacterial suspension of the selected strains *Rhodococcus* 2B23, 2B27 and 3B12 at a concentration of 10^8^ cell/ml and laid on petri dishes containing MS medium supplemented with sucrose. After vernalization, the seeds were put in a growth chamber for three days at 22°C and 50% humidity, then the seedlings were transferred on new petri dishes with the same medium and let grow for seven additional days.

Glass pots containing 0.3 g of quartz sand (SiO_2_ acid washed, Merk) were sterilized and 10 μl of an acetone solution containing 0.5 mg/ml of 4-chlorobiphenyl (PCB No. 3, Alsachim) or 2,2′,5-trichlorobiphenyl (PCB No. 18, Merk) were spiked directly on the sand letting the acetone evaporate, in order to obtain a final quantity of 0.005 mg of PCB in each pot. Then 2.7 g of sterile and dried commercial soil mixture (Vigorplant) was added to the pots, mixed thoroughly with the sand using a sterile glass stick and wet with 5 ml of sterile ½ MS medium. Three ten-days old plantlets of *A*. *thaliana* were transplanted in each pot and watered with additional 1 ml of ½ MS solution.

Five microcosm replicates were setup for each bacterial strain (2B23, 2B27 and 3B12) and PCB congener, together with five replicates of a planted non-inoculated control (NC). A “pristine” planted control without PCB was also setup to verify any effect of the PCB congeners on *A*. *thaliana* growth. The microcosms were incubated for 18 days in a growth chamber at 22°C and 60% humidity and watered every day with 1 ml of ½ MS solution. At the end of the experiment the plants were removed from the pots and shoot fresh and dry weight was measured as described above to evaluate the PGP activity of the inoculated strains. To assess the ability of the inoculated strains to colonize and survive in the rhizosphere environment, three replicates consisting of 0.1 g of roots and adherent rhizosphere soil were randomly picked from each treatment (3B12, 2B23, 2B27 and NC), suspended in 900 μl of 0.9% NaCl physiological solution, smashed with a sterile pestle and vortexed for 15 minutes. The suspension was diluted in ten-fold series and plated on Petri dishes containing agarised mineral medium supplemented with sodium pyruvate 30 mM and biphenyl crystals on the petri lid. The number of colonies attributed to the *Rhodococcus* strains was assessed by counting the yellow colonies due to the production of 2-hydroxy-6-oxo-6-phenylhexa-2,4-dienoic acid (HOPDA) [42]. The identity of the re-isolated bacteria was verified by performing an ITS-PCR fingerprinting [32] on three colonies randomly picked from each inoculation treatment as described above, and by comparing the results with the ITS profile of the strains 2B23, 2B27 and 3B12 grown in pure culture. To compare the number of cells retrieved at the end of the experiment to the one attached on the seed surface at the time of bacterization, the same isolation method was applied to triplicate seeds for each treatment before germination.

## Results

### Isolation and identification of bacterial isolates

One hundred and twenty-eight bacterial strains able to grow on mineral medium supplemented with biphenyl as unique carbon source were obtained from the three-months biostimulated root surrounding soil of *Phalaris arundinacea* subjected to a redox cycle [23]. Sixty-six percent of these strains were originally isolated on biphenyl, 29% on limonene and 5% on naringin, demonstrating that *P*. *arundinacea* rhizosphere potentially enriches cultivable bacteria able to use PSMs, which could act as PCB co-metabolites in the aerobic degradation pathways. After ITS-PCR de-replication, one representative strain from each polymorphic ITS profile (n = 56) was chosen for taxonomic identification through 16S rRNA. The phylogenetic diversity of the collection was limited: the great majority of the strains belonged to the phylum Actinobacteria, while only one isolate was affiliated to Proteobacteria. At the genus level, thirty-one strains (55% of the whole collection) were identified as *Arthrobacter* and four as *Pseudoarthrobacter* (7%). Members of the genera *Gordonia*, *Rhodococcus*, and *Streptomyces* represented together 33% of the collection with six strains each. Two isolates belonged to the genus *Micromonospora* and the only identified *Proteobacteria* strain belonged to the genus *Pseudomonas* (Table 1). The identification of all the strains from each ITS representative at the species level (S1 Table) demonstrates a low level of diversity even at lower ranking. The most represented genus among the collection, *Arthrobacter*, contained isolates belonging only to 2 species *A*. *orizae* (26 isolates) and *A*. *pascens* (5 isolates).

**Table 1:** Taxonomic affiliation at the genus level of each polymorphic ITS representative from the root surrounding soil of *Phalaris arundinacea*.

Genus	N° of strains	%
*Arthrobacter*	31	55%
*Pseudarthrobacter*	4	7%
*Gordonia*	6	11%
*Micromonospora*	2	4%
*Rhodococcus*	6	11%
*Streptomyces*	6	11%
*Pseudomonas*	1	2%

The number of phylogenetically identified strains, one for each polymorphic ITS profile, and the relative percentage in the collection is reported for each bacterial genus.

### *In vitro* characterization of the isolates for their potential to enhance rhizoremediation

**Fig 2:**
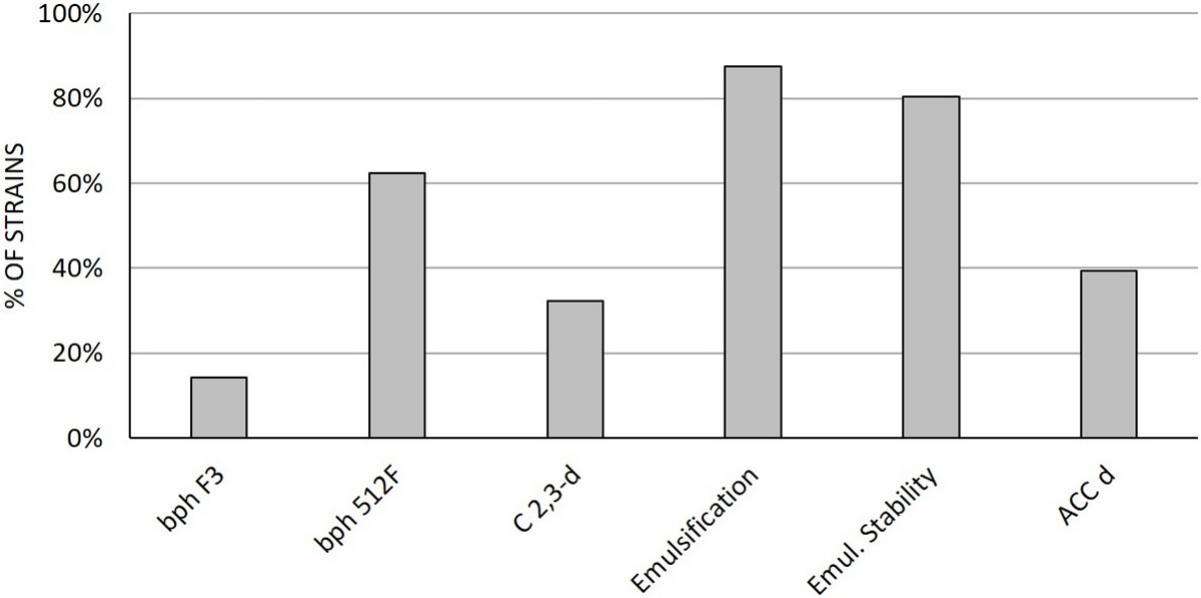
Screening of the bacterial strains for activities related to rhizoremediation potential. The histograms represent the percentage of strains displaying positive results in each test. BphA F3 and 512F = PCR amplification of the *bphA* gene with primers F3/R1 and 512F/674R; C 2,3 D = 2,3-catechol dioxygenase activity; Emul. = emulsification activity; Emul. St. = emulsification stability; ACCd = ACC-deaminase activity.

The screening for activities significant for rhizoremediation was performed *in vitro* on 56 bacterial strains, one for each polymorphic ITS profile detected in the collection (Fig 2 and S1 Table). Thirty-two strains showed catechol dioxygenase activity through meta-cleavage of the aromatic ring, demonstrating the occurrence in the bacteria collection of degrading potential toward aromatic compounds. The majority of the strains (49 out of 56, 88%) was able to produce biosurfactants/bioemulsifiers, performing an emulsification with mineral oil that remained stable after 48 h for the 80% of the isolates.

The 56 bacteria were further characterised *in vitro* for a PGP-related trait of particular interest in phyto/rhizoremediation, *i*.*e*. ACC deaminase activity. This activity was retrieved in 39% of the strains (Fig 2).

The *bphA* gene was detected in the genome of 35 out of 56 strains using primers 512F/674R [34] (S1 Table). All the obtained partial gene sequences showed high nucleotide sequence identity (99%) with *Rhodococcus* sp. *bphA* gene sequences (S2A Table). Additional PCR reaction was then performed on the 35 positive strains (S1 Table) using the F3/R1 primers set [35], aiming to amplify a broader region of the *bphA* gene and to explore its diversity within the collection. However, only *Arthrobacter* sp. strains 2B7, 2B8, *Rhodococcus* spp strains 2B23, 2B27, 3B12, *Streptomyces* spp. 2N21, 2N22, 2N24 displayed *bphA* amplicons of the predicted size (542 base pairs, S1 Table). When we analysed this longer *bphA* gene fragment, only the amplicon sequences of *Rhodococcus* strains were confirmed as *bphA* genes (100% of identity), while sequences obtained from *Arthrobacter* 2B7 and 2B8 and of *Streptomyces* 2N21 showed higher identity to the α-subunit of a generic rieske non-heme iron oxygenase and to 3-phenylpropionate dioxygenase, respectively (S1 Fig and S2B Table). While the presence of the *bphA* gene is considered a proxy for the capacity to degrade PCB [17, 26], the other detected sequences cannot directly be related to a PCB degradation pathway. Hence, we decided to select for subsequent characterization those strains that, according to amplicon sequence, demonstrated to carry biphenyl dioxygenase genes with both the applied primer sets. Besides the three *Rhodococcus* strains, *Arthrobacter* strains 2B7 and 2B8 displaying a generic Rieske non-heme iron oxygenase gene were included in the characterization to assess the possible role of this not clearly identified gene in PCB degradation.

### *In vivo* PGP activity and PCB degradation ability of selected bacterial strains

Strains 2B7, 2B8, 2B23, 2B27 and 3B12, whose *bphA* partial gene sequences were successfully amplified with both primer sets, were selected for an *in vivo* PGP test on the model plant *Arabidopsis thaliana*. All the tested strains demonstrated an influence on the architecture of *A*. *thaliana* plant root system by promoting lateral root development, as shown in Fig 1A for the strain B12. The promotion of root’s growth resulted in a significant increase (from 38% to 83%) of plant fresh weight compared to the non-inoculated control for strains 2B23, 2B27 and 3B12, all belonging to the genus *Rhodococcus*, while no significant differences were observed for *Arthrobacter* strains 2B7 and 2B8 (Fig 1B).

**Fig 3:**
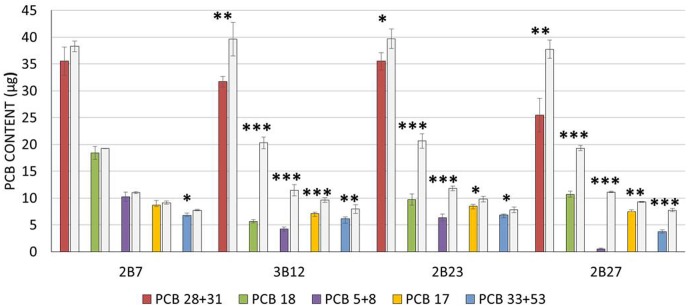
Evaluation of the PCB degradation ability of bacterial strains by resting cell assay. The average contents of the most abundant PCB congeners measured in the flasks is reported for each strain tested. The stars indicate statistically significant depletion when comparing the bacterial strains with their inactivated control (values represented in grey) according to Student T-test (* = p<0.05, ** = p<0.01 *** = p<0.001).

Since we were interested in those strains combining PGP capacity and PCB degradation potential, the strains 3B12, 2B23 and 2B27 were further subjected to a resting cell assay to assess their ability to degrade PCB using the commercial mixture Delor 103. Strains 2B7 and 2B8 were not able to promote plant growth under *in vivo* conditions (Fig 1B), belonged to the same genus and presented a bphA sequence not homologous to the biphenyl dioxygenase gene (S2B Table). We included in the resting cell assay experiment one of these strains (i.e. 2B7), to explore if bacteria harbouring a generic rieske non-heme iron oxygenase could actually perform PCB degradation. A complete list of all the PCB congeners measured after 48 h of incubation is reported in Table 2. The three *Rhodococcus* strains 3B12, 2B23 and 2B27, showed a significant decrease in the total PCB content after 48 h of incubation compared to the autoclaved abiotic controls, demonstrating a degrading ability toward several PCB congeners (Table 3). *Rhodococcus* sp. strain 3B12 also displayed a significant decrease of the total PCB quantity compared to the control strain *Pseudomonas alcaliphila* JAB1 with disrupted *bph* operon (Table 3). *Rhodococcus* strains 3B12, 2B23 and 2B27 showed a significant decrease of all the five most abundant PCB congeners measured, mainly dichloro- (PCB 5+8) and trichlorobiphenyls (PCB 17, PCB 18, PCB 28+31, PCB 33). No depletion in total PCB content was instead observed in bottles inoculated with *Arthrobacter* sp. strain B7 (Table 2 and Fig 3). The identification of the PGP and PCB degradative strains 3B12, 2B23 and 2B27 was confirmed through complete 16S rRNA gene sequencing. The strains presented over 99% of sequence identity and all belonged to the *Rhodococcus jostii* species (S3 Table).

**Table 2:** PCB congener’s quantification calculated as a mean of the replicate serum bottles for each bacterial strain at the end of the resting cell assay.

PCB congener	B7	B7X	B12	B12X	B23	B23X	B27	B27X	JAB1
**28+31**	35,53	38,31	31,69	39,65	35,53	39,72	25,49	37,76	34,29
**18**	18,41	19,25	5,67	20,32	9,73	20,64	10,72	19,32	18,28
**5+8**	10,26	11,01	4,21	11,45	6,36	11,80	0,54	11,14	10,15
**17**	8,72	9,14	7,07	9,64	8,48	9,84	7,48	9,28	8,34
**16**	8,14	8,79	8,31	9,17	9,46	9,50	8,89	8,91	8,52
**33+53**	6,73	7,75	6,11	7,95	6,82	7,83	3,73	7,75	6,51
**22**	5,34	5,73	4,95	6,04	5,47	6,00	5,23	5,75	4,97
**41+64+71+72**	5,21	5,54	4,93	5,49	5,49	5,70	5,58	5,52	4,61
**32**	5,12	5,82	4,71	6,01	5,53	6,01	5,33	5,69	4,98
**66+95**	4,62	4,70	4,03	4,81	4,19	4,83	3,57	4,95	3,95
**4**	4,06	4,28	1,83	4,61	2,81	4,45	4,38	4,12	4,09
**48+47**	4,04	4,26	3,94	4,37	4,34	4,51	2,84	4,27	3,96
**49**	3,99	4,22	3,90	4,58	4,41	4,53	4,47	4,21	3,83
**70+76**	3,47	3,54	3,06	3,73	3,16	3,77	3,60	3,77	2,92
**44**	3,44	3,66	2,47	3,84	3,19	3,98	3,27	3,67	3,30
**52**	3,32	3,58	3,26	3,67	3,65	3,67	3,64	3,60	3,21
**42**	3,17	3,54	3,11	3,49	3,59	3,63	3,49	3,46	3,12
**56+60**	3,15	3,12	2,74	3,17	2,86	3,32	2,45	2,93	2,72
**37**	3,05	2,98	2,23	2,99	2,28	2,68	3,09	3,35	2,13
**15**	2,32	2,55	1,79	2,46	2,09	2,38	1,88	2,38	2,00
**19**	2,04	2,13	1,98	2,11	2,20	2,14	2,25	2,18	1,91
**74**	1,76	1,76	1,58	1,85	1,61	1,79	1,78	1,87	1,48
**25+26**	1,55	1,69	0,62	1,73	0,66	1,74	1,57	1,57	1,39
**45**	1,49	1,56	1,48	1,57	1,76	1,62	0,69	1,64	1,42
**40**	1,29	1,34	1,24	1,34	1,29	1,44	1,38	1,44	1,24
**27+24**	1,00	1,07	0,40	1,18	0,60	1,19	0,78	1,16	1,02
**6**	0,98	1,16	0,21	1,14	0,43	1,09	0,24	1,04	1,06
**110**	0,44	0,39	0,34	0,40	0,33	0,45	0,52	0,47	0,34
**118**	0,32	0,17	0,20	0,23	0,19	0,26	0,26	0,31	0,18
**99+113**	0,27	0,26	0,23	0,27	0,24	0,28	0,31	0,27	0,25
**101+90**	0,27	0,26	0,24	0,26	0,26	0,29	0,29	0,27	0,23
**97**	0,19	0,17	0,16	0,22	0,16	0,25	0,27	0,30	0,15
**84+92+89**	0,18	0,19	0,19	0,27	0,32	0,23	0,15	0,15	0,27
**63**	0,16	0,15	0,13	0,15	0,13	0,17	0,20	0,22	0,15
**87**	0,02	0,01	0,01	0,02	0,01	0,02	0,02	0,02	0,02
**Tot. PCB (Sum)**	**154,05**	**164,10**	**119,03**	**170,16**	**139,66**	**171,72**	**120,36**	**164,76**	**146,97**

The table reports the average PCB content (μg) measured for each bacterial strain and the inactivated controls. The PCB congeners were measured in each replicate serum bottle after 48 h of incubation with the PCB mixture Delor 103 and the bacterial suspension. X = inactivated control represented by the autoclaved strain culture. JAB1 = *Pseudomonas* sp. strain JAB1

**Table 3:** Average total PCB contents calculated as a sum of the different congeners measured in each replicate serum bottle.

**Sample**	**B7**	**B7X**	**B12**	**B12X**	**B23**	**B23X**	**B27**	**B27X**
**Average tot. PCB content (μg)**	154,05	164,10	119,03	170,16	139,66	171,72	120,36	164,76
**Standard deviation**	10,73	2,30	5,84	12,89	5,97	8,60	14,16	13,79
**p (Student T test)**	0,18515		**0,00077**		**0,00544**		**0,00145**	
**Sample**	**B7**	**JAB1**	**B12**	**JAB1**	**B23**	**JAB1**	**B27**	**JAB1**
**Average tot. PCB content (μg)**	154,05	146,97	119,03	146,97	139,66	146,97	120,36	146,97
**Standard deviation**	10,73	4,75	5,84	4,75	5,97	4,75	14,16	4,75
**p (Student T test)**	0,42958		**0,00175**		0,21999		0,11465	

p values of the statistical analysis comparing each strain with its inactivated control (“X” samples) and the control strain JAB1 are reported and significant difference values (p<0.05) are highlighted in bold.

### Evaluation of bioaugmentation potential in microcosm conditions

**Fig 4:**
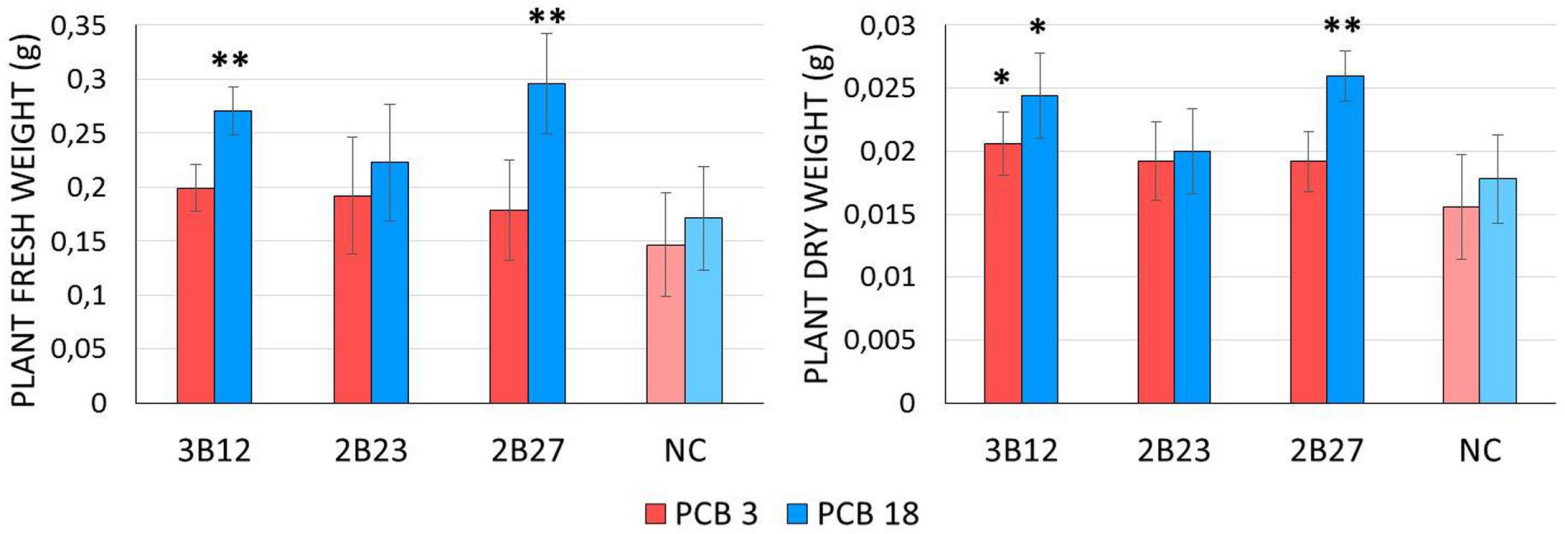
Plant growth promoting ability in microcosm conditions. Screened strains and plant fresh and dry weight measurement results are reported on the X- and Y-axes, respectively. Different colours represent different PCB congeners used to spike the soil. The stars indicate statistically significant differences compared to the negative control (* = p<0.05, ** = p<0.01).

To evaluate the bioaugmentation potential of the three *Rhodococcus* strains that showed significant PCB reduction in the resting cell assay, we set up a simulated bioagumentation treatment by seed priming of the model plant *A*. *thaliana* in a soil artificially contaminated with two different PCB congeners. After three weeks of growth plants that were subjected to seed priming with two out of the three bacterial strains showed increased fresh and dry shoot biomass compared to the non-inoculated controls, particularly with the highest chlorinated congener, PCB18 (2,2′,5-PCB, 2,2′,5-Trichlorobiphenyl) (Fig 4). According to Student T-test, plants inoculated with strains 3B12 and 2B27 and growing on soil spiked with PCB 18 showed fresh and dry weight values significantly higher compared to the non-inoculated control (p<0.01). In addition, treatment 3B12 significantly increased plant shoot dry weight (p<0.05) also in soil spiked with PCB 3 (4-Chlorobiphenyl). At the end of the experiment, all the three *Rhodococcus* strains were successfully isolated from the rhizospheric soil and roots of *A*. *thaliana*, at a concentration of 10^8^ CFU per gram of soil / root biomass for each plant (S2A Fig). Considering that we estimated a starting concentration of bacterial cells of about 10^4^ cell per seed (S4 Table) on primed seed before germination, the 4-fold increase in bacterial counts proved the ability of the strains to actively colonize the root system and duplicate in the rhizosphere. No colonies displaying the HOPDA yellow halo [42] were retrieved from the root system of non inoculated plants, leading to speculate that all the colonies retrieved from bacterized plants belonged to the strains used in seed priming. This was confirmed by the results of the ITS-PCR performed on the re-isolated strains (S2B Fig).

## Discussion

From the historically contaminated SIN-Brescia-Caffaro soil biostimulated for three months by the plant *P*. *arundinacea*, we obtained a collection of aerobic bacterial strains able to use PSMs and/or biphenyl as unique carbon source. All the isolates, with the exception of one *Pseudomonas* strain, were affiliated to the phylum Actinobacteria. The phylogenetic composition of this collection differs from a previous bacterial collection isolated from spontaneous plants naturally selected in the same area of the SIN Caffaro, which comprised a higher phylogenetic diversity and, besides Actinobacteria (up to 75% of the isolates obtained from the same plant), contained also Proteobacteria (up to 32%) and Bacilli (up to 13%) [43]. This apparent discrepancy can be explained by the combination of the selective isolation conditions applied in this work, which exploited PSMs as unique carbon source, and the different origin of the root-associated soil, sampled from potted *P*. *arundinacea* subjected to repeated flooding to induce cyclic redox conditions. Actinobacteria, a significant part of spontaneous plants rhizosphere in the SIN-Brescia Caffaro as also demonstrated by cultivation independent methods [43], showed to be an important potential degradative phylogenetic group in this aged PCB contaminated site, and its dominance in plant soil surrounding root suggests its keystone role, being specifically enriched by rhizo-stimulation. Further experiments are nevertheless needed to sustain this speculation. Bacteria belonging to the Actinobacteria class have been previously detected in PCB polluted soils [29] and are known for their metabolic versatility and the potential for bioremediation of persistent organic pollutants, including PCBs [43–45]. More than half of the strains isolated in this study harboured the *bphA* gene that is essential to begin the aerobic pathway responsible for PCB degradation [46]. Furthermore, one third of the strains were able to cleave catechol, one of the most common intermediates in the degradative pathways of aromatic compounds, including pollutants and secondary plant metabolites. Hence, its cleavage is an important activity essential for the mineralization of these compounds [47]. Coherently with the different phylogenetic composition of the isolate collections, also the affiliation of *bphA* partial gene sequences provided different results from our previous findings on spontaneous plants associated bacteria [43]. All the *bphA* related sequences amplified from strains isolated from *P*. *arundinacea* root-surrounding soil clustered with *Rhodococcus*-like sequences and none of the sequences clustered with *Pseudomonas*-like ones that were retrieved in the spontaneous plant rhizosphere microbiota. However, even though 35 out of 56 strains displayed *Rhodococcus*-like partial *bphA* sequences when amplified with primers 512F/674R, the further amplification of a longer gene fragment with F3/R1 primer set was successful only for eight strains among 35, and amplicon sequencing revealed a broader sequence diversity. In fact, we identified three different types of Rieske non-heme dioxygenases in strains belonging to different genera (*Arthrobacter* sp. 2B7 and 2B8, *Rhodococcus* sp. 3B12, 2B23 and 2B27, and *Streptomyces* sp. 2N21) and confirmed the identity with biphenyl dioxygenase sequences only for *Rhodococcus* strains 2B23, 2B27 and 3B12. This result conforms to previous studies on the great diversity of enzymes included in this family, nevertheless not always related to PCB degradation [35]. *Arthrobacter* sp. 2B7, whose *bphA* sequence was identified as a generic Rieske non-heme dioxygenase, confirmed these findings since it did not show any significant PCB degradation potential when tested in resting cell assay.

Besides having degrading potential, the majority of the collection showed additional traits of interest for PCB bioremediation. Most of the strains produced a stable emulsification with mineral oil, a result that suggests their potential capacity to increase hydrophobic contaminant bioavailability for degradation [48]. The emulsification capacity is a common trait among the class Actinobacteria, in particular the genera *Arthrobacter*, *Gordonia* and *Rhodococcus* are known to produce molecules able to disperse organic pollutant contaminations [48]. This aspect is pivotal also for the remediation of PCB-polluted soils, since one of the main limits to the biodegradation of these molecules is that they are highly hydrophobic and tightly bound to the soil organic matter [49], a feature particularly relevant in aged-contaminated soils. ACC deaminase activity was detected in all the isolates affiliated to the genus *Streptomyces*, in twelve *Arthrobacter* and five *Pseudoarthrobacter* strains. This activity is a well known direct PGP mechanism by which bacteria can help plants to counteract the environmental stress (*e*.*g*. soil phytotoxicity) [15] by reducing the level of the stress-related phytohormone ethylene, which precursor is ACC.

Three *Rhodococcus* sp. strains harbouring a *bph*A homologous gene and two *Arthrobacter* sp. strains harbouring a non-heme iron oxygenase homologous gene (S2 Table) were selected for subsequent *in vivo* evaluation. *Arthrobacter* sp. strains did not show *in vivo* PGP activity on *A*. *thaliana*, and the strain 2B7, selected for the resting cell assay, did not show PCB degrading ability, indicating that the non-heme iron oxygenase homologous genes were not related to PCB degrading potential. On the contrary, all of the *Rhodococcus* sp. isolates, further identified as belonging to the *R*. *jostii* species, significantly promoted the growth of *A*. *thaliana* and induced lateral root development. This is a relevant ability for rhizoremediation, as a wider extension of the plant root system is a highly desirable trait that would result in an increase of the soil volume subjected to the influence of rhizodepositions [50]. The PGP activity of these strains was confirmed even in artificially contaminated soil, and their ability to colonize the plant rhizosphere reaching high population density and to increase the plant shoot biomass was demonstrated on *A*. *thaliana* plants exposed to different PCB congeners. Interestingly, the same *Rhodococcus* strains also displayed a significant ability of PCB depletion in resting cell assay, making them ideal candidates for rhizoremediation. This result is in accordance with previously published data reporting different *Rhodococcus* strains as PCB degraders and as rhizosphere-associated bacteria [29, 42, 51]. Other authors highlighted, moreover, that *A*. *thaliana* root exudates enhanced the *bphA* gene expression and PCB degradation by *Rhodococcus erythropolis* strain U23A [17, 51], providing further evidence of the activity of this genus induced by rhizo-remediation. Considering that the *Rhodococcus* strains isolated in this work demonstrated to stably colonize the root system of *A*. *thaliana* and to promote its development, we can infer the occurrence of a bacteria-plant beneficial interaction that, once confirmed in other plant species, may be exploitable for rhizoremediation strategies of PCB polluted soils. Indeed, we recently demonstrated in different plant species the pivotal role of plant-microbiota interaction to decrease the concentrations of several PCB congeners from the SIN Brescia-Caffaro soil under semi-field conditions. *P*. *arundinacea* plants subjected to redox cycling demonstrated in particular to induce in 18 months the significant reduction of 20 PCB congeners [23]. Overall, our findings reveal the potential of *Rhodococcus* strains to sustain plant growth and PCB biodegradation in a historically polluted soil like that of the SIN Brescia-Caffaro. The effectiveness of these strains in terms of plant growth promotion and PCB mineralization will be further assessed at mesocosm level, to fully evaluate their actual possibilities of application in the field.

## Conclusion

We established from plant-biostimulated soil a collection of Actinobacteria able to use PSMs and biphenyl as unique carbon source, displaying different activities that suggest their potential to enhance PCB rhizoremediation. The amplification of the *bphA* genes using two different primer sets provided different results in terms of detection and identification of the gene fragments, as longer amplicons reduced the number of positive strains detected and, at the same time, uncovered a broader gene diversity. PCB degradation assays further demonstrated that amplicons with nucleotide sequence not homologous to the *bphA* gene do not seem to be correlated with the PCB degradation pathway. All in all, our results highlight that further studies on biphenyl dioxygenase gene diversity and functionality are still necessary to consider the presence of the *bph*A gene as a robust indicator of bacterial PCB degradation ability. Three *Rhodococcus* strains showed both the abilities to promote plant root development in the model plant *A*. *thaliana* and to degrade PCBs, making them suitable candidate to sustain PCB rhizoremediation through a site-tailored bioaugmentation approach. Since *A*. *thaliana* root exudates previously showed to promote PCB degradation by a *Rhodoccoccus* bacterial strain, our results open future research perspectives on the study of this plant-bacteria interaction for PCB rhizoremediation purposes. Further investigation should be directed to test these strains *in vivo* on plants of interest for rhizoremediation and to better characterize their PCB degradation activity.

## Supporting information

S1 Table Taxonomic identification and *in vitro* screening of activities linked to the rhizoremediation potential of all the strains of the PCR-ITS de-replicated collection (n = 56). Strains were identified by 16S rRNA gene amplification, partial gene sequencing and the use of the EZBioCloud identification service. Co. % = sequence completeness in percentage; Si. %. = percentage of similarity with the reference strain. Gray squares indicate positive results. Nd = not determined. Bph F3 and 512F = PCR amplification of the *bphA* gene with primers F3/R1 and 512F/674R, respectively; C 2,3 D = 2,3-catechol dioxygenase activity; Emul. = emulsification activity; Emul. St. = emulsification stability; ACCd = ACC-deaminase activity.
(DOCX)

S2 Table Identification of the *bphA* partial nucleotide sequences according to the NCBI database. **A.** PCR products amplified with primers 512F and 674R. **B.** PCR products amplified with primers F3 and R1 (see the Material and Methods section for further details on the PCR protocols).
(DOCX)

S3 Table Taxonomic identification of the strains 3B12, 2B23 and 2B27 through complete 16S rRNA gene sequencing and analysis using the EzBioCloud database. (DOCX)

S4 Table Estimation of bacterial cells number attached to the seed surface after seed priming. The table reports the mean values of CFU isolated from triplicate seeds for each bacterial treatment.
(DOCX)

S1 Fig Phylogenetic tree of the putative *bphA* gene sequences amplified with primers F3 and R1 from the isolated strains 2B23, 2B27, 3B12, 2B7, 2B8 and 2N21. (TIF)

S2 Fig Root system colonization assay. **A.** The bars represent the mean values of colonies forming units (CFU) calculated per gram of soil and root biomass of triplicate plants for each of the three strains 3B12, 2B23 and 2B27. No colonies were retrieved in the non inoculated control (NC). **B.** Gel electrophoresis of the ITS-PCR performed on three colonies randomly picked after re-isolation from each inoculation treatment. NC indicates the PCR negative control, PC indicates the positive control performed on the DNA extracted from the pure-culture strains.
(TIF)
