# Supplementary material for: Novel PCB-degrading Rhodococcus strains able to promote plant growth for assisted rhizoremediation of historically polluted soils
Source: PLoS One. 2019 Aug 22;14(8):e0221253. doi: 10.1371/journal.pone.0221253 (PMC6705854; doi:10.1371/journal.pone.0221253)
Supplement: S1 Table — Strains were identified by 16S rRNA gene amplification, partial gene sequencing and the use of the EZBioCloud identification service. Co. % = sequence completeness in percentage; Si. %. = percentage of similarity with the reference strain. Gray squares indicate positive results. Nd = not determined. Bph F3 and 512F = PCR amplification of the bphA gene with primers F3/R1 and 512F/674R, respectively; C 2,3 D = 2,3-catechol dioxygenase activity; Emul. = emulsification activity; Emul. St. = emulsification stability; ACCd = ACC-deaminase activity. (DOCX) [file pone.0221253.s001.docx]

|  |  | **Isolate identification** | | |  | **Rhizoremediation pot.** | | | | | |
| --- | --- | --- | --- | --- | --- | --- | --- | --- | --- | --- | --- |
| **Strain** | **Co. %** | **Identification** | **Si. %** | **Accession N°** |  | **bph F3** | **bph 512F** | **C 2,3-d** | **Emul.** | **Emul. St.** | **ACC d** |
| 1B1 | 58.8 | *Gordonia amicalis* | 100 | BANS01000072 |  |  |  |  |  |  |  |
| 1B10 | 58.5 | *Arthrobacter oryzae* | 100 | CLG_48533 |  |  |  |  |  |  |  |
| 1B16 | 55.1 | *Arthrobacter oryzae* | 99.25 | CLG_48533 |  |  |  |  |  |  |  |
| 1B18 | 56.1 | *Arthrobacter oryzae* | 99.75 | CLG_48533 |  |  |  |  |  |  |  |
| 1B21 | 61.8 | *Micromonospora chalcea* | 99.66 | X92594 |  |  |  | nd | nd | nd | nd |
| 1B23 | 67.5 | *Arthrobacter oryzae* | 99.69 | CLG_48533 |  |  |  |  |  |  |  |
| 1B3 | 55.2 | *Gordonia amicalis* | 100 | BANS01000072 |  |  |  |  |  |  |  |
| 1B4 | 59.3 | *Gordonia hongkongensis* | 100 | LC072670 |  |  |  |  |  |  |  |
| 1B9 | 64.6 | *Arthrobacter oryzae* | 98.93 | CLG_48533 |  |  |  |  |  |  |  |
| 1L10 | 63.3 | *Gordonia hongkongensis* | 99.78 | LC072670 |  |  |  |  |  |  |  |
| 1L11 | 61.8 | *Gordonia hongkongensis* | 99.78 | LC072670 |  |  |  |  |  |  |  |
| 1L16 | 57.8 | *Arthrobacter pascens* | 99.28 | X80740 |  |  |  |  |  |  |  |
| 1L20 | 66.0 | *Rhodococcus aetherivorans* | 99.44 | AF447391 |  |  |  |  |  |  |  |
| 1L5 | 55.9 | *Streptomyces viridochromogenes* | 99.38 | [AB184728](https://www.ezbiocloud.net/16SrRNA?ac=AB184728) |  |  |  |  |  |  |  |
| 1N10 | 57.4 | *Streptomyces ambofaciens* | 99.76 | CP012382 |  |  |  |  |  |  |  |
| 1N31 | 64.2 | *Gordonia amicalis* | 99.46 | BANS01000072 |  |  |  |  |  |  |  |
| 2B10 | 62.6 | *Arthrobacter oryzae* | 100 | CLG_48533 |  |  |  |  |  |  |  |
| 2B13 | 60.0 | *Rhodococcus ruber* | 99.54 | LRRL01000064 |  |  |  |  |  |  |  |
| 2B15 | 63.1 | *Pseudarthrobacter sulfonivorans* | 99.67 | AF235091 |  |  |  |  |  |  |  |
| 2B16 | 61.4 | *Arthrobacter oryzae* | 99.66 | CLG_48533 |  |  |  |  |  |  |  |
| 2B17 | 59.0 | *Pseudarthrobacter sulfonivorans* | 99.65 | AF235091 |  |  |  |  |  |  |  |
| 2B19 | 58.5 | *Arthrobacter oryzae* | 99.88 | CLG_48533 |  |  |  |  |  |  |  |
| 2B2 | 61.6 | *Arthrobacter oryzae* | 99.89 | CLG_48533 |  |  |  |  |  |  |  |
| 2B23 | 72.9 | *Rhodococcus jostii* | 99.62 | FNTL01000001 |  |  |  |  |  |  |  |
| 2B27 | 65.3 | *Rhodococcus wratislaviensis* | 99.68 | BAWF01000105 |  |  |  |  |  |  |  |
| 2B28 | 62.9 | *Arthrobacter pascens* | 100 | CLG_48533 |  |  |  |  |  |  |  |
| 2B29 | 57.7 | *Arthrobacter oryzae* | 99.88 | CLG_48533 |  |  |  |  |  |  |  |

|  |  | **Isolate identification** | | |  | **Rhizoremediation pot.** | | | | | |
| --- | --- | --- | --- | --- | --- | --- | --- | --- | --- | --- | --- |
| **Strain** | **Co. %** | **Identification** | **Si. %** | **Accession N°** |  | **bph F3** | **bph 512F** | **C 2,3-d** | **Emul.** | **Emul. St.** | **ACC d** |
| 2B3 | 59.2 | *Arthrobacter oryzae* | 99.53 | CLG_48533 |  |  |  |  |  |  |  |
| 2B30 | 66.6 | *Arthrobacter oryzae* | 99.69 | CLG_48533 |  |  |  |  |  |  |  |
| 2B5 | 57.3 | *Arthrobacter oryzae* | 99.88 | CLG_48533 |  |  |  |  |  |  |  |
| 2B7 | 57.2 | *Arthrobacter oryzae* | 100 | CLG_48533 |  |  |  |  |  |  |  |
| 2B8 | 55.3 | *Arthrobacter oryzae* | 99.75 | CLG_48533 |  |  |  |  |  |  |  |
| 2L10 | 64.3 | *Arthrobacter oryzae* | 99.89 | CLG_48533 |  |  |  |  |  |  |  |
| 2L21 | 63.8 | *Streptomyces albogriseolus* | 100 | AJ494865 |  |  |  |  |  |  |  |
| 2L22 | 54.0 | *Arthrobacter oryzae* | 100 | CLG_48533 |  |  |  |  |  |  |  |
| 2L27 | 59.3 | *Arthrobacter oryzae* | 99.88 | CLG_48533 |  |  |  |  |  |  |  |
| 2L29 | 54.5 | *Arthrobacter pascens* | 99.23 | X80740 |  |  |  |  |  |  |  |
| 2L30 | 59.5 | *Arthrobacter oryzae* | 100 | CLG_48533 |  |  |  |  |  |  |  |
| 2N20 | 67.9 | *Pseudarthrobacter siccitolerans* | 89.57 | CAQI01000001 |  |  |  |  |  |  |  |
| 2N21 | 61.6 | *Streptomyces ossamyceticus* | 100 | LIQX01000228 |  |  |  |  |  |  |  |
| 2N22 | 62.2 | *Streptomyces ossamyceticus* | 100 | LIQX01000228 |  |  |  |  |  |  |  |
| 2N24 | 56.5 | *Streptomyces ossamyceticus* | 100 | LIQX01000228 |  |  |  |  |  |  |  |
| 3B10 | 63.4 | *Rhodococcus ruber* | 99.78 | LRRL01000064 |  |  |  |  |  |  |  |
| 3B12 | 70.2 | *Rhodococcus jostii* | 99.60 | FNTL01000001 |  |  |  |  |  |  |  |
| 3B14 | 57.8 | *Micromonospora aurantiaca* | 100 | [CP002162](https://www.ezbiocloud.net/16SrRNA?ac=CP002162) |  |  |  | nd | nd | nd | nd |
| 3B15 | 57.7 | [*Arthrobacter oryzae*](https://www.ezbiocloud.net/taxonomy?tn=Arthrobacter%20oryzae) | 100 | CLG_48533 |  |  |  |  |  |  |  |
| 3B20 | 63.6 | *Pseudarthrobacter sulfonivorans* | 99.78 | AF235091 |  |  |  |  |  |  |  |
| 3B21 | 58.8 | *Arthrobacter oryzae* | 99.88 | CLG_48533 |  |  |  |  |  |  |  |
| 3B23 | 59.3 | *Arthrobacter oryzae* | 99.88 | CLG_48533 |  |  |  |  |  |  |  |
| 3B26 | 64.2 | *Arthrobacter pascens* | 99.89 | X80740 |  |  |  |  |  |  |  |
| 3B3 | 58.6 | *Arthrobacter oryzae* | 99.88 | CLG_48533 |  |  |  |  |  |  |  |
| 3B4 | 63.0 | *Arthrobacter oryzae* | 100 | CLG_48533 |  |  |  |  |  |  |  |
| 3B5 | 63.9 | *Arthrobacter oryzae* | 100 | CLG_48533 |  |  |  |  |  |  |  |
| 3B7 | 55.4 | *Arthrobacter pascens* | 98.88 | X80740 |  |  |  |  |  |  |  |
| 3B8 | 61.2 | *Arthrobacter oryzae* | 100 | CLG_48533 |  |  |  |  |  |  |  |
| 3L27 | 71.1 | *Pseudomonas hunanensis* | 99.81 | JX545210 |  |  |  |  |  |  |  |
