# Supplementary material for: Novel PCB-degrading Rhodococcus strains able to promote plant growth for assisted rhizoremediation of historically polluted soils
Source: PLoS One. 2019 Aug 22;14(8):e0221253. doi: 10.1371/journal.pone.0221253 (PMC6705854; doi:10.1371/journal.pone.0221253)
Supplement: S2 Table — A. PCR products amplified with primers 512F and 674R. B. PCR products amplified with primers F3 and R1 (see the Material and Methods section for further details on the PCR protocols). (DOCX) [file pone.0221253.s002.docx]

| **A. Primers 512F/674R** | | | |
| --- | --- | --- | --- |
| **Strain** | **Closest relative** | **Acc. N°** | **Id. %** |
| **1B3** | Rhodococcus wratislaviensis strain P13 biphenyl 2,3-dioxygenase alpha subunit (bphA1) gene | KP972446 | 98 |
| **1B10** | Rhodococcus wratislaviensis strain P13 biphenyl 2,3-dioxygenase alpha subunit (bphA1) gene | KP972446 | 99 |
| **1L11** | Rhodococcus wratislaviensis strain G10 putative Rieske non-heme iron oxygenase alpha subunit gene | KP972448 | 100 |
| **2B3** | Rhodococcus wratislaviensis strain P13 biphenyl 2,3-dioxygenase alpha subunit (bphA1) gene | KP972446 | 99 |
| **2B7** | Rhodococcus wratislaviensis strain G10 putative Rieske non-heme iron oxygenase alpha subunit gene | KP972448 | 100 |
| **2B8** | Rhodococcus wratislaviensis strain G10 putative Rieske non-heme iron oxygenase alpha subunit gene | KP972448 | 100 |
| **2B13** | Rhodococcus wratislaviensis strain G10 putative Rieske non-heme iron oxygenase alpha subunit gene | KP972448 | 100 |
| **2B23** | Rhodococcus wratislaviensis strain P13 biphenyl 2,3-dioxygenase alpha subunit (bphA1) gene | KP972446 | 99 |
| **2B27** | Rhodococcus wratislaviensis strain P13 biphenyl 2,3-dioxygenase alpha subunit (bphA1) gene | KP972446 | 99 |
| **2B28** | Rhodococcus wratislaviensis strain G10 putative Rieske non-heme iron oxygenase alpha subunit gene | KP972448 | 99 |
| **2L29** | Rhodococcus wratislaviensis strain P13 biphenyl 2,3-dioxygenase alpha subunit (bphA1) gene | KP972446 | 98 |
| **2N22** | Rhodococcus wratislaviensis strain G10 putative Rieske non-heme iron oxygenase alpha subunit gene | KP972448 | 100 |
| **2N24** | Rhodococcus wratislaviensis strain G10 putative Rieske non-heme iron oxygenase alpha subunit gene | KP972448 | 100 |
| **3B3** | Rhodococcus wratislaviensis strain G10 putative Rieske non-heme iron oxygenase alpha subunit gene | KP972448 | 100 |
| **3B8** | Rhodococcus wratislaviensis strain P13 biphenyl 2,3-dioxygenase alpha subunit (bphA1) gene | KP972446 | 98 |
| **3B10** | Rhodococcus wratislaviensis strain P13 biphenyl 2,3-dioxygenase alpha subunit (bphA1) gene | KP972446 | 99 |
| **3B12** | Rhodococcus wratislaviensis strain P13 biphenyl 2,3-dioxygenase alpha subunit (bphA1) gene | KP972446 | 99 |
| **LB400** | Paraburkholderia xenovorans partial bphA gene for biphenyl dioxygenase alpha subunit, isolate LB400 | LT840239 | 99 |
| **B. Primers F3/R1** | | | |
|  |  |  |  |
| **Strain** | **Closest relative** | **Acc. N°** | **Id. %** |
| **2B23** | Uncultured Rhodococcus sp. clone NS7 biphenyl 2,3-dioxygenase alpha subunit (bphAa) gene | JN675900 | 100 |
| **2B27** | Uncultured Rhodococcus sp. clone NS7 biphenyl 2,3-dioxygenase alpha subunit (bphAa) gene | JN675900 | 100 |
| **3B12** | Uncultured Rhodococcus sp. clone NS7 biphenyl 2,3-dioxygenase alpha subunit (bphAa) gene | JN675900 | 100 |
| **2B7** | Arthrobacter sp. 3YC3 putative Rieske non-heme iron oxygenase alpha subunit gene | DQ166965 | 97 |
| **2B8** | Arthrobacter sp. 3YC3 putative Rieske non-heme iron oxygenase alpha subunit gene | DQ166965 | 99 |
| **2N21** | Streptomyces scabiei 3-phenylpropionate dioxygenase alpha subunit | FN554889 | 90 |
| **LB400** | Paraburkholderia xenovorans LB400 chromosome 3, complete sequence, gene bphA | CP008761 | 100 |
