# Supplementary material for: Novel PCB-degrading Rhodococcus strains able to promote plant growth for assisted rhizoremediation of historically polluted soils
Source: PLoS One. 2019 Aug 22;14(8):e0221253. doi: 10.1371/journal.pone.0221253 (PMC6705854; doi:10.1371/journal.pone.0221253)
Supplement: S3 Table — (DOCX) [file pone.0221253.s003.docx]

| **Strain** | **Completeness (%)** | **Identification** | **Similarity (%)** | **Accession N°** |
| --- | --- | --- | --- | --- |
| 3B12 | 96.5 | *Rhodococcus jostii* | 99.78 | FNTL01000001 |
| 2B23 | 96.5 | *Rhodococcus jostii* | 99.86 | FNTL01000001 |
| 2B27 | 94.9 | *Rhodococcus jostii* | 99.85 | FNTL01000001 |
