# Supplementary material for: Novel PCB-degrading Rhodococcus strains able to promote plant growth for assisted rhizoremediation of historically polluted soils
Source: PLoS One. 2019 Aug 22;14(8):e0221253. doi: 10.1371/journal.pone.0221253 (PMC6705854; doi:10.1371/journal.pone.0221253)
Supplement: S4 Table — The table reports the mean values of CFU isolated from triplicate seeds for each bacterial treatment. (DOCX) [file pone.0221253.s004.docx]

| **Strain** | **colonies N** | **CFU/seed** | **St. Dev.** |
| --- | --- | --- | --- |
| **3B12** | 16,4 | 1,64E+04 | 1,54E+04 |
| **2B23** | 21,9 | 2,19E+04 | 1,81E+04 |
| **2B27** | 15,9 | 1,26E+04 | 1,55E+04 |
