# Supplementary figures and images for: Novel PCB-degrading Rhodococcus strains able to promote plant growth for assisted rhizoremediation of historically polluted soils
Source: PLoS One. 2019 Aug 22;14(8):e0221253. doi: 10.1371/journal.pone.0221253 (PMC6705854; doi:10.1371/journal.pone.0221253)

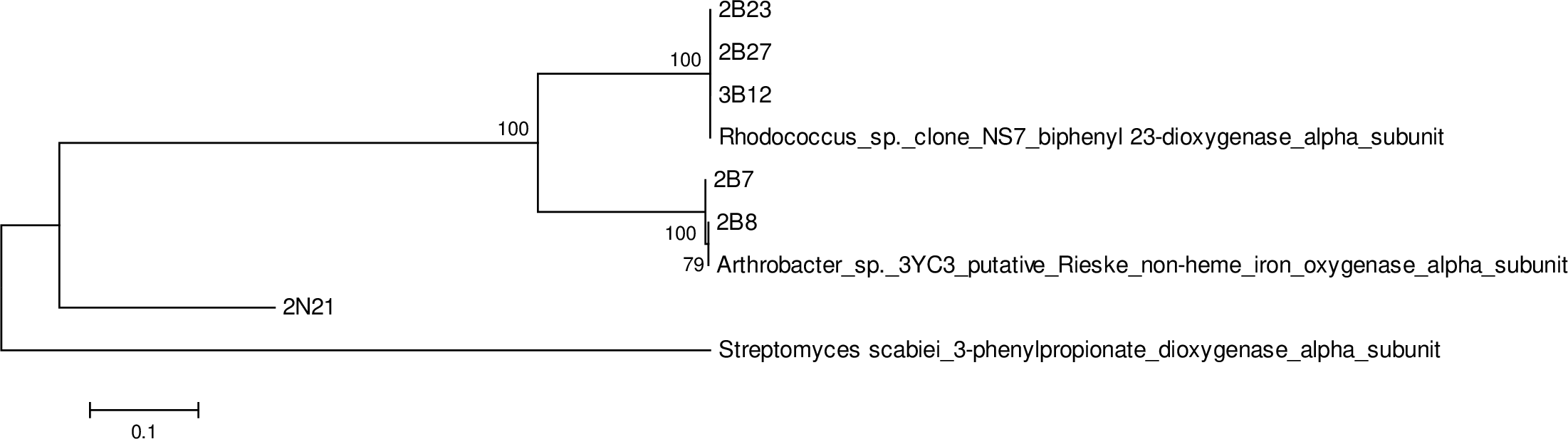

Supplement: S1 Fig — (TIF) [file pone.0221253.s005.tif]

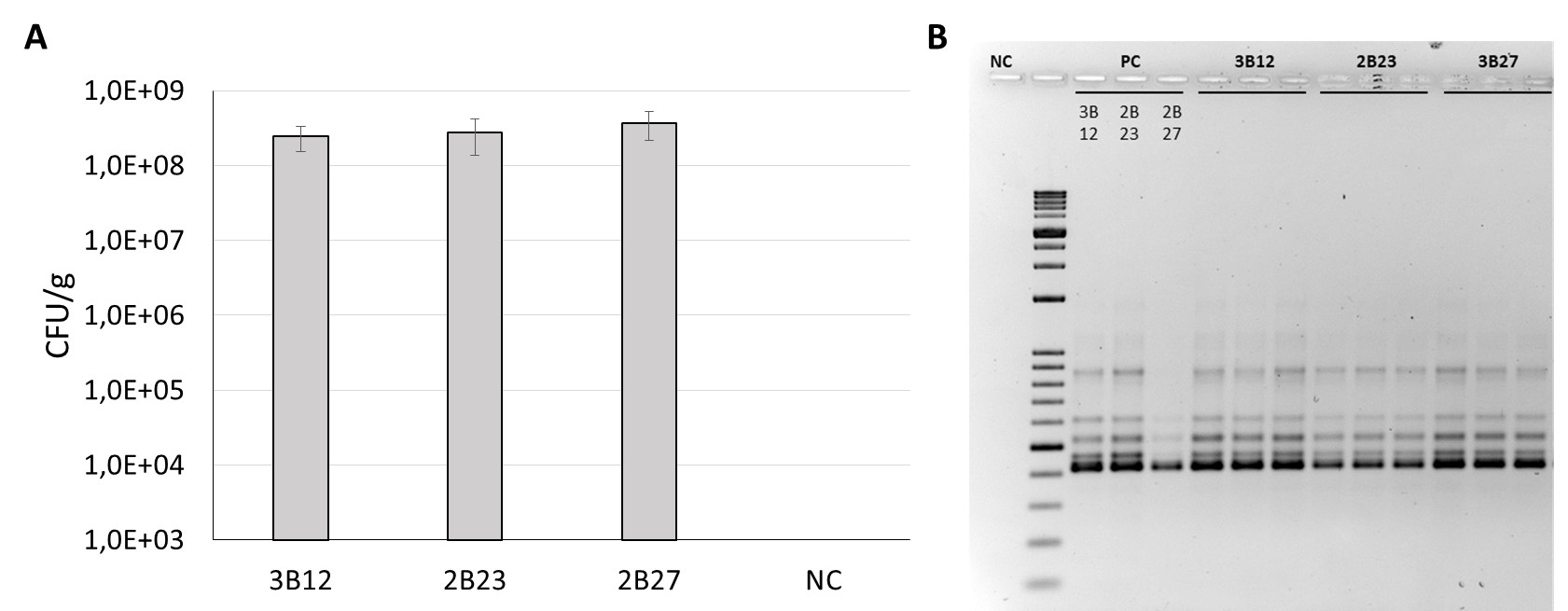

Supplement: S2 Fig — A. The bars represent the mean values of colonies forming units (CFU) calculated per gram of soil and root biomass of triplicate plants for each of the three strains 3B12, 2B23 and 2B27. No colonies were retrieved in the non inoculated control (NC). B. Gel electrophoresis of the ITS-PCR performed on three colonies randomly picked after re-isolation from each inoculation treatment. NC indicates the PCR negative control, PC indicates the positive control performed on the DNA extracted from the pure-culture strains. (TIF) [file pone.0221253.s006.tif]
